# Carers’ experiences, needs and preferences during inpatient stroke rehabilitation: a protocol for a systematic review of qualitative studies

**DOI:** 10.1186/s13643-015-0097-0

**Published:** 2015-08-11

**Authors:** Julie A. Luker, Susanne Bernhardsson, Elizabeth Lynch, Carolyn Murray, Olivia P. Hill, Julie Bernhardt

**Affiliations:** International Centre for Allied Health Evidence and the Sansom Institute, University of South Australia, Adelaide, Australia; Florey Institute of Neuroscience & Mental Health, University of Melbourne, Melbourne, Australia; Division of Physiotherapy, Department of Medical and Health Sciences, Linköping University, Linköping, Sweden; Närhälsan Hönö/Öckerö Rehabilitation, Region Västra Götaland, Strandvägen 35, 475 40 Hönö, Sweden

**Keywords:** Stroke, Rehabilitation, Carers, Qualitative research, Systematic review

## Abstract

**Background:**

Large numbers of people provide carer roles for survivors of stroke. Person-centred stroke rehabilitation must consider the perspectives of carers, as stroke affects not only the stroke survivor but also the quality of life and health of the carers. There is little collective knowledge about stroke carers’ experiences, needs and preferences during the inpatient stroke rehabilitation process to then inform person-centred service improvements.

Our objective is to report and synthesise experiences, needs and preferences of the carers of stroke survivors undergoing rehabilitation in inpatient settings.

**Methods/design:**

We will conduct a systematic review of qualitative studies using a thematic synthesis methodology.

We will follow the Enhancing Transparency in Reporting the Synthesis of Qualitative Research Guidelines (ENTREQ) and search the following databases for relevant articles: MEDLINE, Cumulative Index to Nursing and Allied Health Literature (CINAHL), PsycINFO, Embase, and Web of Science. No language or publication date constraints will be applied. Eligible studies will have to use qualitative methods of data collection and analysis and reported data from the carers of stroke survivors who underwent inpatient stroke rehabilitation. Studies will be eligible for inclusion if they report the experiences, needs and preferences of carers regarding inpatient rehabilitation environments, organisation, care systems, therapeutic interventions, information exchange, carer training, discharge and community service planning and other issues of relevance to their roles as carers. Study selection and assessment of quality will be performed independently by two reviewers. Any disagreement will be resolved by a third reviewer. Data will be extracted by one reviewer, tabled, and checked for accuracy by another reviewer. All text reported in studies’ results, discussion and conclusion sections will be entered into the NVivo software for analysis. Extracted texts will be inductively coded independently by two reviewers and analysed in three phases using thematic synthesis. Descriptive and analytical themes will be developed.

**Discussion:**

This study is expected to provide new insights into the perspectives of stroke survivors’ carers. Increased knowledge about carer perspectives and preferences will inform person-centred improvements in stroke rehabilitation.

**Study registration:**

PROSPERO registration number: CRD42015017315.

**Electronic supplementary material:**

The online version of this article (doi:10.1186/s13643-015-0097-0) contains supplementary material, which is available to authorized users.

## Background

Due to the high prevalence of stroke worldwide and the associated long-term disabilities, large numbers of people take on a role of a caregiver for someone who has survived a stroke. Approximately 50 % of stroke survivors will require support from a carer [1]. In 2009, the Australian Survey of Disability, Ageing and Carers identified 26,367 Australians who cared for people with stroke as their main condition [2].

The definition of ‘carer’ varies across stroke studies [3], with these people also known as lay, informal, unpaid, primary, family carers or caregivers (called carers from this point). In the context of stroke management, carers may be family members or friends who provide physical, practical or emotional help to someone after their stroke [4]. Carers may initially be involved as supportive, concerned hospital visitors or take a more active role in inpatient management and rehabilitation. Following the stroke survivor’s discharge back into the community, they may take on complex carer roles, providing a broad range of physical and psychosocial support. The time commitments of carers also vary greatly from around the clock, live-in duties to occasional visits or phone contacts and may be solo roles or shared with others. In Australia, 68 % of carers spent 40 h or more per week caring for people with stroke [2].

Carers can be seen both as colleagues of health professionals, in contributing to decision making and support for the patient, and as health service consumers themselves having their own problems and special needs [5]. It is well recognised that carers, like the stroke survivors, experience high levels of burden, isolation and frustration which can significantly impact on their physical and mental health status and quality of life [3, 6–10].

A qualitative systematic review of the experiences, perspectives and preferences of stroke survivors undergoing inpatient rehabilitation identified important information to guide improvements in stroke services [11]. Carer engagement was valued by stroke survivors during rehabilitation and may contribute to progress in recovery. Stroke survivors reported several issues that could be influenced by their carers during the inpatient period, such as the alleviation of boredom and loneliness, assistance with functional task practice or exercise, encouragement of motivation and self-efficacy and information gathering.

Our work will be underpinned by concepts of person-centred care as defined by the Australian Commission on Safety and Quality in Health Care as “… an approach to the planning, delivery, and evaluation of health care that is grounded in mutually beneficial partnerships among health care providers, patients, and families” [12]. In keeping with a person-centred approach, the needs and goals of stroke survivors and their carers are at the core of the stroke rehabilitation process [13, 14]. Truly, person-centred stroke rehabilitation must take into account the requirements of carers as they adapt to their changing life circumstances and prepare for their new caregiving role during hospitalisation and after inpatient discharge. Carers report that the most valuable services to assist them to cope in the long term are those provided while still in hospital [8]. However, these services do not always adequately prepare carers [15].

Following return to the community, when inpatient support systems are no longer available, carers may experience great difficulties accessing the information, skills, strategies and supports they need for their new caregiving roles [16, 17]. Carers are a diverse group, and they need ongoing emotional support, information and training [18]. When this is inadequate, the mental and physical health of carers may decline [19].

The best ways to provide preparatory training and support for the new role of a carer are still unclear. Currently, there is little collective knowledge about stroke carers’ experiences, needs and preferences during the inpatient stroke rehabilitation process.

### Objectives

- The primary objective of this systematic review will be to synthesise and report the experiences, needs and preferences of carers of stroke survivors undergoing rehabilitation in inpatient settings.
- The secondary objective will be to deliver evidence-informed recommendations for person-centred inpatient stroke rehabilitation that include consideration of, and planning for, the needs and the contribution of carers.

## Methods/design

This systematic review has been registered with the International Prospective Register of Systematic Reviews (PROSPERO): CRD42015017315.

### Study design

This review will follow the ENTREQ statement (Enhancing Transparency in Reporting the Synthesis of Qualitative Research) in reporting the stages of our qualitative synthesis [20]. While similarities exist between ENTREQ and Preferred Reporting Items for Systemic Reviews and Meta-Analyses (PRISMA) guidelines, the latter relate primarily to quantitative reviews [21]. A thematic synthesis methodology will be employed, as described by Thomas and Harden [22] and detailed below.

### Study eligibility criteria

SPICE question structure:

- Setting: inpatient stroke rehabilitation facilities
- Perspective: carers of stroke survivors
- Intervention: interventions for stroke survivors and/or their carers
- Comparison: n/a
- Evaluation: the experiences, needs and preferences of carers

### Study design

Studies will be included if they used established qualitative methods of data collection (interviews, focus groups, direct observation, action research or questionnaires that allowed free text) and qualitative methods of analysis.

Studies will be excluded if they are mixed methods studies where the qualitative data cannot be separated out or studies with mixed participant groups or various settings where data for the carers of stroke survivors cannot be separated out. Conference abstracts and opinion pieces will not be considered. Studies will also be excluded based on poor quality and must meet at least two of the four appraisal criteria developed by Carroll and colleagues [23].

### Study participants

Studies will be included if data were obtained directly from people who are the carers of a person with stroke, where the person with stroke underwent rehabilitation in an inpatient setting.

We define carers as the spouse or partner, family members, friends or ‘significant others’ who provide physical, practical or emotional support to someone after their stroke. There will be no other restrictions applied to these carers (such as live-in versus visiting carers, receiving government carers’ allowance or not).

### Intervention/exposure

Included studies will consider the process of stroke rehabilitation as it affects the carers of people with stroke. This may include, but is not limited to, rehabilitation environments, the organisation and systems of rehabilitative care, therapeutic interventions, information exchange, carer training and planning for discharge. We define stroke rehabilitation as:

…a dynamic, progressive, goal orientated process aimed at enabling a person with impairment to reach their optimal physical, cognitive, emotional, communicative and/or social functional level ([24], p. 4).

### Study settings

Studies will be included if carers provide information related to their experiences, needs or preferences during the stroke survivor’s time in inpatient rehabilitation settings. These settings may include tertiary hospitals where rehabilitation commences in the acute phase, sub-acute rehabilitation units, and rehabilitation units within nursing homes that exist in some countries.

### Search strategy

We will search the databases that are considered relevant data sources for pertinent studies: MEDLINE, Cumulative Index to Nursing and Allied Health Literature (CINAHL), PsycINFO, Embase, and Web of Science. A search strategy will be developed on MEDLINE and then adapted for the other databases. This will include medical subject headings (MeSH) and free-text terms using applicable controlled vocabulary (see examples shown in Additional file 1). No language or publication date constraints will be applied. Reference lists of included studies and relevant systematic reviews will be hand searched to identify additional studies for potential inclusion.

### Selection of studies

Search results will be entered into Endnote folders, and any duplicates and clearly irrelevant titles will be removed. Study selection will occur in two phases, each of which assesses potential studies against the review’s criteria. Each selection phase will be conducted independently by two reviewers, who will then meet to compare results. The preliminary screening phase will assess titles and abstracts. Studies will be excluded at this phase if both reviewers agree to exclude, but title/abstracts without consensus agreement will go on to full-text screening. During full-text screening, consensus must be reached to include or exclude studies from the review. If necessary, a third reviewer will make the final decision. A flow diagram will report the selection process and reasons for exclusion, as suggested by PRISMA guidelines [21].

#### Non-English studies

Where database searches (conducted with English search terms) find non-English studies that meet our inclusion criteria, attempts will be made to translate the publications prior to data extraction and analysis. We acknowledge that meaning attached to language may be lost in translated papers, and this will be discussed as a limitation of our review findings. To avoid potentially confounding our synthesised results, we will report the translated studies separately.

### Quality appraisal

#### Preliminary assessment

To assist the internal validity of this review, included studies must meet at least two of the four quality reporting criteria developed by Carroll and colleagues [23] regarding study design, selection of participants, methods of data collection and analysis. The characteristics of studies excluded on the basis of quality will be reported, but their data will not be included in thematic synthesis analysis.

#### Comprehensive assessment

Included studies will be independently assessed by two reviewers for comprehensive and explicit reporting using the COREQ 32-item checklist [20]. Assessment findings will be discussed, and consensus will be reached on scoring. A third reviewer will make the final decision if agreement cannot be reached. Assessment findings will be presented in a table for easy comparison across studies.

### Data extraction

Data on the characteristics of included studies will be extracted from the entire documents and entered into a purpose-built datasheet by one reviewer and checked for accuracy by another. Extracted fields will be reported in a table and include referencing details, theoretical framework, methodological approach, aims of the study, sample size, participant characteristics, participants’ relationship to the stroke survivor, additional participant groups, data collection method, analysis method and study setting/country. All data presented as text in the ‘Results/findings’, ‘Discussion’ and ‘Conclusion’ sections of papers will be extracted and entered into the NVivo 10 software [25] to assist data management and analysis.

### Analysis

A thematic synthesis approach will be undertaken [22]. Using this transparent analytical method, the development of descriptive themes will allow the review to remain ‘close’ to the primary studies. The subsequent analytical themes will be derived through a stage of interpretation which enables analysis to ‘go beyond’ the primary studies and generate new explanations or hypotheses relevant to the review’s aims.

Coding and thematic development will be conducted in three rigorous stages, each involving independent consideration by two or more researchers, discussions and consensus. In the first stage of analysis, codes will be inductively derived from the data in an iterative process of attributing codes to small sections of meaning within the text, moving back and forward across studies and constantly comparing data and codes. One reviewer will code and manage the data using the NVivo software, while a second reviewer will independently code on hard copies. In the second stage, the reviewers will work collaboratively, to group codes into logical and meaningful clusters in a hierarchical tree structure, forming descriptive themes and sub-themes. Finally, analytical themes relating to the aims of the review will be developed. These will deliver evidence-informed recommendations for person-centred inpatient stroke rehabilitation that include consideration of the needs of carers. The descriptive and analytical themes will be reported along with supporting quotations from the original studies. This will be examined by all reviewers, and a final version agreed.

The concepts of *carer* and the roles of carers may differ between cultures. To facilitate the international transferability of our findings, we will be mindful of this as we analyse the data and will report findings within cultural subsets if appropriate.

## Discussion

Evidence derived from the carers of stroke survivors can inform person-centred improvements in stroke rehabilitation. We will deliver evidence-informed recommendations for person-centred inpatient stroke rehabilitation that include consideration of the experiences, needs and preferences of carers. These recommendations may improve the preparation of carers for their new roles, as stroke survivors’ transition from inpatient rehabilitation to home. These improvements may subsequently influence carers’ ability to manage care provision, as well as the sustainability of their relationship with the stroke survivors. To our knowledge, this will be the first study to systematically synthesis information derived directly from carers regarding their experiences, needs and preferences during the inpatient rehabilitation phase of stroke recovery.

### Limitations and strengths

As with all reviews, the findings will be dependent on the quality of the original studies. Quality appraisal for qualitative studies is notoriously difficult to perform, and there is a risk that the inclusion of data from flawed studies may bias our findings [20]. To minimise this, we will exclude studies with inadequate reporting [23]. As discussed earlier, the translation of non-English studies may result in the loss of the intended meaning. So that readers can form their own conclusions regarding the generalizability and trustworthiness of our findings, the comprehensiveness of the report in all included studies will be described, and translated studies will be reported separately.

The review quality will be strengthened by the involvement of multiple reviewers at all stages of the review process, including a consumer representative reviewer, to ensure consensus, consistency and a person-centred focus. Our chosen analytical approach, thematic synthesis, is a tested method that preserves a clear and transparent connection between the findings and text of the primary studies and the themes and conclusions of the review. These are principles traditionally valued in high-quality systematic reviews. The review findings will be distributed and made publically available in peer-reviewed publications and presentations.

## Additional file

Additional file 1:
**Search strategy for Ovid Medline database**. Example of the database search strategy developed for Ovid Medline.

## Abbreviations

COREQ: consolidated criteria for reporting qualitative research
ENTREQ: Enhancing Transparency in Reporting the Synthesis of Qualitative Research
MeSH: medical subject headings
PRISMA: Preferred Reporting Items for Systemic Reviews and Meta-Analyses
SPICE: setting, perspective, intervention, comparison, evaluation
