# Supplementary material for: Carers’ experiences, needs and preferences during inpatient stroke rehabilitation: a protocol for a systematic review of qualitative studies
Source: Syst Rev. 2015 Aug 11;4:108. doi: 10.1186/s13643-015-0097-0 (PMC4532249; doi:10.1186/s13643-015-0097-0)
Supplement: Additional file 1: — Search strategy for Ovid Medline database. Example of the database search strategy developed for Ovid Medline. [file 13643_2015_97_MOESM1_ESM.docx]

**Additional file 1: Search strategy for Ovid Medline**

Carers’ experiences, needs and preferences during inpatient stroke rehabilitation: a systematic review of qualitative studies.

PECO:

Participants: carers of stroke survivors

Exposure: inpatient stroke rehabilitation

Control: n/a

Outcomes: the experiences, needs and preferences of carers

| *Search term* | |
| --- | --- |
| **Population** | |
|  | exp Stroke/ |
|  | exp Family/ or famil*.mp. |
|  | hospital volunteers.mp. or Hospital Volunteers/ |
|  | visitors to patients.mp. or exp Visitors to Patients/ |
|  | Carer*.mp. |
|  | caregivers.mp. or exp Caregivers/ |
|  | exp Friends/ or friend$1.mp |
|  | adult children.mp. or exp Adult Children/ |
|  | siblings.mp. or exp Siblings/ |
|  | spouse.mp. or exp Spouses/ |
|  | (partner$1 or husband$1 or wi$3 or defacto).mp. |
|  | 2 or 3 or 4 or 5 or 6 or 7 or 8 or 9 or 10 or 11 |
|  | 12 AND 1 |
| **Exposure** | |
|  | exp Rehabilitation Centers/ or exp Rehabilitation/ or rehabilitat*.mp. or exp Rehabilitation Nursing/ |
|  | (hospitali* or in?patient$1).mp. |
|  | 14 or 15 |
|  | 13 AND 16 |
| **Study Design** | |
|  | Qualitative Research/ |
|  | Cohort Studies/ |
|  | Observational Study/ |
|  | Focus Groups/ |
| 22. | Interview, Psychological/ or Interview/ |
| 23. | ((semi-structured or semistructured or unstructured or informal or in-depth or indepth or face-to-face or structure or guide) adj3 (interview* or discussion* or question?aire*)).mp. |
| 24. | (ethnograph* or fieldwork or 'field work' or 'key informant').mp. |
| 25 | 18 or 19 or 20 or 21 or 22 or 23 or 24 |
| 26 | 17 AND 26 |
